# Tin Selenide (SnSe): Growth, Properties, and Applications

**DOI:** 10.1002/advs.201700602

**Published:** 2018-01-08

**Authors:** Weiran Shi, Minxuan Gao, Jinping Wei, Jianfeng Gao, Chenwei Fan, Eric Ashalley, Handong Li, Zhiming Wang

**Affiliations:** ^1^ Institute of Fundamental and Frontier Sciences University of Electronic Science and Technology of China Chengdu 610054 P. R. China; ^2^ Institute of Fundamental and Frontier Sciences University of Electronic Science and Technology of China Chengdu 610054 P. R. China; ^3^ State Key Laboratory of Electronic Thin Films and Integrated Devices School of Microelectronics and Solid‐State Electronics University of Electronic Science and Technology of China Chengdu 610054 P. R. China

**Keywords:** doping, growth, optoelectronics, thermoelectric materials, tin selenide

## Abstract

The indirect bandgap semiconductor tin selenide (SnSe) has been a research hotspot in the thermoelectric fields since a *ZT* (figure of merit) value of 2.6 at 923 K in SnSe single crystals along the *b*‐axis is reported. SnSe has also been extensively studied in the photovoltaic (PV) application for its extraordinary advantages including excellent optoelectronic properties, absence of toxicity, cheap raw materials, and relative abundance. Moreover, the thermoelectric and optoelectronic properties of SnSe can be regulated by the structural transformation and appropriate doping. Here, the studies in SnSe research, from its evolution to till now, are reviewed. The growth, characterization, and recent developments in SnSe research are discussed. The most popular growth techniques that have been used to prepare SnSe materials are discussed in detail with their recent progress. Important phenomena in the growth of SnSe as well as the problems remaining for future study are discussed. The applications of SnSe in the PV fields, Li‐ion batteries, and other emerging fields are also discussed.

## 1 Introduction

Tin‐based binary chalcogenide compounds Sn—X (X = S, Se, Te) have been explored due to their potential applications in the next generation electronic, optical, optoelectronic, and flexible systems. For instance, tin sulfide (SnS) and tin telluride (SnTe) are promising in the application of solar cells.^1^, ^2^ Among the Sn—X materials family, tin sulfide (SnS) and tin selenide (SnSe) are the materials consisting nontoxic and economical earth‐abundant elements, which significantly promote their value in sustainable electronic and photonic systems.^3^, ^4^, ^5^

The Sn—X compounds are typical layered materials and commonly crystallize into three phases: hexagonal, monoclinic, and orthorhombic; hexagonal and monoclinic phases are denoted by chemical formula SnX_2_, and orthorhombic is denoted by SnX. The crystal structure of SnX_2_ is quite similar to that of layered molybdenum chalcogenides, a prototype 2D‐material system. Compared to thin‐layered molybdenum chalcogenides, thin‐layered SnX_2_ is expected to possess superior electronic and optoelectronic properties. Due to this feature, SnX_2_ has been extensively studied during recent years. An excellent review dealing with growth and device applications of SnX_2_ can be found,^6^ with emphasis on low‐dimensional 2D structures.

As compared to SnX_2_ the SnX materials have been less studied although they were also proposed to be candidate materials for photovoltaic and optoelectronic applications with proper energy band structures and excellent electronic properties.^1^, ^7^ The research enthusiasms on SnX compounds did not arise until SnSe was revealed to act as a superior thermoelectric (TE) material owning a dimensionless figure of merit (*ZT*) value of 2.6 along a specific crystalline orientation (*b*‐axis) by Zhao et al.^8^ It is quite stimulating because the energy conversion efficiency of a TE system with *ZT* value greater than 3 will surpass that of a traditional heat engine.^9^ Many researchers have also focused on the ways of further enhancing the efficiency, i.e., by means of metal‐doping, hole‐doping, and type transitions.^10^, ^11^, ^12^, ^13^

The layered orthorhombic structure of SnSe can be regarded as a distorted NaCl‐type structure. This structure leads to the high Grüneisen parameters, which results in the anharmonic and anisotropic bonding. The ultralow thermal conductivity of orthorhombic SnSe originates from the unique bonding nature of the crystal.^8^ Large cubic structured SnSe (SnSe‐CUB), which is supposed to be as stable as orthorhombic SnSe, has also been identified in nanocrystalline materials^14^ and in thin films^15^ recently. In addition, the large cubic tin sulfide–tin selenide thin film stacks reported in 2016 are also promising in photovoltaic and thermoelectric applications.^16^

Despite SnSe has become more and more popular recently, there is no comprehensive review fully focusing on SnSe yet. Here, we summarize the research achievements and progress in SnSe material as a timely reference to the readers. Initially, the review will deal with the intriguing physical properties of SnSe (crystal structures and band structures), followed by the various methods ever employed for the synthesis of phase‐pure SnSe like hot injection, facile surfactant‐free synthesis, thermal evaporation, insert gas condensation, etc. The important growth parameters based on the previous reports of SnSe single crystals, thin films, and nanostructures have been discussed. Afterwards, electronic, thermoelectric, and optoelectronic characteristics of bulk crystals, thin films, and nanostructures of SnSe will be examined in detail followed by novel applications of SnSe in photovoltaic field, rechargeable batteries, supercapacitors, phase‐change memory devices, and topological insulator (TI). This review will motivate the readers to further boost the investigation on SnSe compound for various research and application purposes. In the following content, unless specifically stated, SnSe is normally regarded as a material with orthorhombic structure.

## 2 Structure and Bonding

### 2.1 Crystal Structure of SnSe

#### 2.1.1 Basic Structure Introduction

The valence electronic configurations of tin (Sn) and selenium (Se) atoms are 4d^10^5s^2^5p^2^ and 4s^2^4p^4^, respectively (for chalcogens it is *n*s^2^ *n*p^4^ where *n* is the periodic number of the element). As the electronegativity of Se atoms is stronger than Sn atoms, Se captures two electrons from the Sn atom which leads to the change in the electronic configuration of Sn from 4d^10^5s^2^5p^2^ to 4d^10^5s^2^5p^0^ and that of Se to 4s^2^4p^6^.^17^ The sp^2^ hybridization results in crumpled surface and each atom forms three covalent bonds with the other three atoms.^18^ As a result, Sn acquires the oxidation state of II because the two 5p electrons are engaged in bonding while the 5s^2^ electrons occupy an inert orbital. However, the Sn (II) lone pair in the electronic structure of Sn(5s) compounds has a great influence on distorting the crystal structure. And the antibonding Sn(5s)‐anion p combination, which is created by the interaction of Sn(5s) lone pair and the anion, supports the coupling of Sn(5s) and Sn(5p) leading to an active asymmetric density. From oxygen (O), sulfur (S), selenium (Se) to tellurium (Te), higher valence p states of the anion correspond to less coupling of Sn(5p) and Sn(5s), yielding less active asymmetric density; this decides the distorting level of electronic structure. Consequently, the herzenbergite structure, which does not fit the strong asymmetry on Sn in tin(II) oxide (SnO), adapts well to the Sn in tin sulfide (SnS) and SnSe.^19^

**Figure 1:**
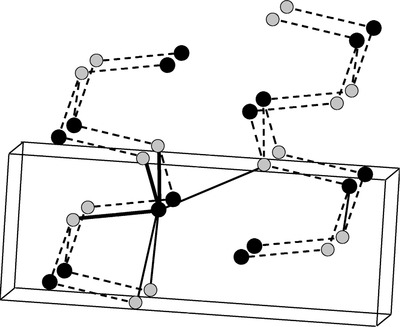
SnS or SnSe atomic structure. Shaded balls stand for selenium and bold ones stand for tin. Dotted lines correspond to bonds and bold ones represent the bonds from one tin atom. Reproduced with permission.^17^ Copyright 1998, American Physical Society.

The Sn(5s) lone pair can also influence the layer separation indirectly. The unit cell contains eight atoms arranged in two adjacent layers which are perpendicular to the longest axis.^20^ The local‐atomic arrangement of tin atoms looks like a distorted octahedron (see **Figure** **1**, 17). Each atom forms six dominant heteropolar bonds, three nearest and strongest bonds of which are with nearest neighbors residing in the same double layers. The two second nearest ones are further neighbors in the same double layers, the sixth nearest neighboring atom lies in the next double layers, and the double layers are linked by the bonds.^21^ Bond lengths of the six heteropolar bonds in SnSe reported by various authors are recorded in **Table** **1**.

**Table 1:** Bond lengths (Å) of the six heteropolar bonds in SnSe reported by various workers

Bond				Ref.
Sn(1)Se	Sn(2)Se	Sn(2)Se	Sn(1)Se	
2.74	2.79	3.34	3.47	17
2.77	2.82	3.35	3.47	21

Some other studies hold the view that one long Sn—X bond and two Sn—Sn bonds supply the bond between double layers.^17^

**Figure 2:**
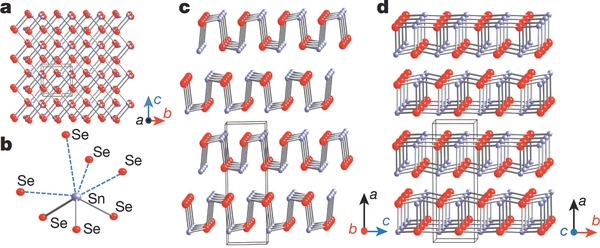
a) Crystal structure along the *a*‐axis: grey, Sn atoms; red, Se atoms. b) Highly distorted SnSe_7_ coordination polyhedron with three short and four long Sn—Se bonds. c) Structure along the *b*‐axis. d) Structure along the *c*‐axis. Reproduced with permission.^8^ Copyright 2014, Nature Publishing Group.

SnSe bulk structure demonstrates a layered orthorhombic structure having space group P_nma_. The structure consists of strongly bound double layers and can be regarded as a distorted rocksalt phase.^19^ Moreover, its perspective views are different along the *a*, *b*, and *c* axial directions, which lead to its anisotropic nature. There are SnSe slabs with a thickness of nearly two‐atoms with strong Sn—Se bonds within the plane of slabs, i.e., along *b*–*c* plane and weaker along *a*‐axis. The perspective view along the *b*‐axis is a zigzag accordion like projection but along the *c*‐axis is an armchair form.^8^, ^22^ Scientists have detected high *ZT* values along the *b*‐axis near and above the transition temperature of about 800 K at which the structure converts from space group of P_nma_ to C_mcm_, with the bandgap changing from 0.61 to 0.39 eV,^23^ and the lattice parameters changing from *a* = 11.49 Å, *b* = 4.44 Å, *c* = 4.135 Å to *a* = 4.31 Å, *b* = 11.70 Å, *c* = 4.31 Å.^1^ The highly distorted SnSe_7_ coordination polyhedral contained in SnSe crystal has three short and four long Sn—Se bonds, as shown in **Figure** **2**.^8^

The cubic binary phase of SnSe identified recently presents a cubic geometry with lattice parameter 11.9702 Å. There are 64 atoms (32 Sn atoms and 32 Se atoms) per unit cell in the model of cubic phase SnSe. This new cubic phase belongs to a class of non‐centrosymmetric crystals, which may lead to the discovery of some interesting and potentially useful properties.^14^

#### 2.1.2 Pressure‐Driven Structure Transition

Pioneering studies have found several approaches to optimize the thermoelectric performance of SnSe, for example, changing the temperature dependence from semiconductive‐like to metallic‐like by doping.^24^ The definite changes in the free carrier concentration and mobility under structure transition provide a better way to modulate the transport properties of SnSe. And recent analysis has indicated that high pressure is a significant factor to promote the structural transitions.

Yan et al.^24^ recently carried out an experiment on pressure‐driven structure transition in orthorhombic SnSe, in which the high pressure was generated by a nonmagnetic diamond anvil cell with an anvil culet of 300 mm in diameter. The conclusion and result of experiment were consistent with the result of first‐principles band structure calculations.

The X‐ray diffraction (XRD) measurement shows the structure properties of SnSe under various pressure conditions of SnSe,^25^ and the XRD pattern of this experiment shows that three new diffraction peaks at about 13.71°, 14.31°, and 24.21° suddenly emerge with pressure gradually increasing to 12.1 GPa, where the phase I–II structural transitions can be clearly observed. The phase II structure can remain with the pressure less than 26.2 GPa. In the decompression process, the whole diffraction peaks of phase I appear again, on the contrary the diffraction peaks of phase II disappear, revealing that the structural transition is reversible. The phases I and II mentioned above are orthorhombic and monoclinic structures of SnSe, respectively.^24^

As for the electrical transport parameters related to the thermoelectric performance of SnSe, electrical resistivity (ρ) and carrier concentration (*n*) have a significant decrease, whereas the carrier mobility (μ) is improved by more than ten times with the pressure increased from ambient to 12.2 GPa. It has been revealed that the pressure level at ≈12.2 GPa is an abnormal variation point, where carrier concentration (*n*) inverts its pressure dependence increased up to 25 GPa and carrier mobility (μ) shows a sharp decrease from nearly 100 cm^2^ V^−1^ s^−1^ at about 12.2 GPa to 10 cm^2^ V^−1^ s^−1^ at 25 GPa. Therefore, smooth drop in electrical resistivity (ρ) from ≈2.82 × 10^−3^ to ≈1.86 × 10^−3^ Ω cm is observed when pressure increases from ≈12.2 to 25 GPa.^24^

It can be summarized that SnSe becomes semimetallic at about 12.6 GPa, followed by the transition from orthorhombic to monoclinic structure, which leads to the variation in carrier concentration and mobility.

### 2.2 The Origin of Ultralow Thermal Conductivity of SnSe

**Figure 3:**
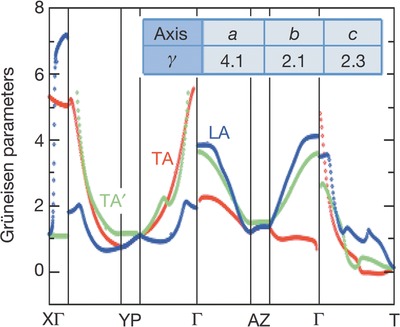
Grüneisen dispersion. Inset: the average Grüneisen parameters along *a‐*, *b*‐, and *c*‐axes. TA, red color; TA′, green color; LA, blue colour. Reproduced with permission.^8^ Copyright 2014, Nature Publishing Group.

A high value of Grüneisen parameters of SnSe at 923 K has been reported by Zhao et al.^8^ Grüneisen dispersions along three directions are shown in **Figure** **3**.^8^ The unexpectedly high Grüneisen parameter with average value of 4.1 along *a*‐axis corresponds to the special crystal structure of SnSe discussed above, which includes the distorted Sn coordination environment and a zigzag accordion‐like geometry of slabs in the *b*–*c* plane.^8^ The zigzag geometry is very flexible and is able to be deformed without changing the SnSe bond length under limited pressure, which dissipates the transportation of the lateral phonons, resulting in high Grüneisen parameter.

The strong lattice anharmonicity which can be estimated from the structure‐related high Grüneisen parameter leads to the ultralow thermal conductivity of 0.0064 compared with 1.48 (silicon), 0.67 (Sn), or 0.02 W cm^−1^ K^−1^ for lead telluride (PbTe), a common thermoelectric material. The anharmonicity can also be shown in the phonons in SnSe with strong anisotropic and giant anharmonicity, which is also related to the resonantly bonding Se p‐states and active Sn lone pair. In simple words, the anisotropic, low thermal conductivity results from the layered structure and strongly anharmonic phonons, both of which are concerned with the Sn lone pair.^26^

### 2.3 Band Structure of SnSe

One of the common characteristic of IV–VI compounds is the narrow bandgap. The bandgap and optoelectronic properties of SnSe have been studied, which illustrate the potential of the ultrathin SnSe nanosheets (NSs) in photodetector and photovoltaic fields.^27^ SnS is similar to SnSe for they both have narrow bandgaps and SnS is excellent for the application in thin‐film solar cells because of its optical bandgap of 1.3 eV and high optical absorption coefficient. Germanium selenide (GeSe) and SnSe crystallize in a similar layered structure but the fundamental bandgap is direct in single layer and double layer GeSe, whereas indirect in SnSe with few‐layers.^22^ Dimensions of these materials affect their electronic structure and physical properties. There are experiments that demonstrate different shapes of p‐type IV–VI semiconductor SnSe material with different bandgaps. The direct and indirect bandgaps are 0.90 and 1.30 eV, respectively, for bulk SnSe. However, as shown in **Table** **2**, the direct bandgap demonstrated by thin films is greater than that of bulk as a result of quantum confinement effect.^28^ In cubic SnSe (SnSe‐CUB), the obtained optical bandgap of ≈1.4 eV is larger than that of bulk orthorhombic SnSe.^15^, ^16^

**Table 2:** Bandgaps (eV) of different shaped SnSe materials

	Single layer	Double layer	Bulk	Nanocolumns
Direct bandgap	1.66	1.62	1.3	
Indirect bandgap	1.63	1.47	0.9	0.93
	Nanosheets (NSs)	Nanoflowers (NFs)	Nanocrystals (NCs)	Nanoplates
Direct bandgap	1.10	1.05	1.71	
Indirect bandgap	0.86	0.95		0.96

**Figure 4:**
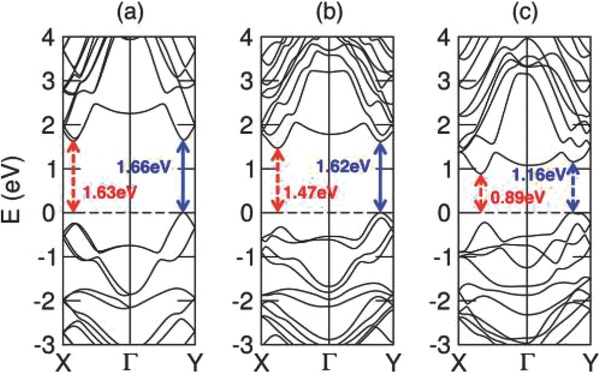
Band structures of a) single‐layer, b) double‐layer, and c) bulk SnSe. Solid arrows indicate direct transitions, and dashed arrows indicate indirect transitions. Reproduced with permission.^22^ Copyright 2015, American Chemical Society.

The energy difference between two conduction‐band minima along Γ–X and Γ–Y between the direct bandgap and indirect bandgap of the single layer material is small (see **Figure** **4**, 22). This also shows that bandgap can be transformed from direct to indirect or vice versa.

As reported in the literature, the bandgap of SnSe nanoplates is 0.96 eV which is significantly blue‐shifted from that of the bulk SnSe (0.9 eV). However, the bandgap of nanocolumns is red‐shifted to 0.93 eV in comparison with nanoplates.^29^ By conducting the transformation way of Kubelka–Munk, the direct and indirect bandgaps of SnSe NSs were extrapolated to be 1.10 and 0.86 eV, and the direct and indirect bandgaps of SnSe nanoflowers (NFs) were determined to be 1.05 and 0.95 eV, respectively.^27^ As for typical SnSe nanowires, it was found to exhibit indirect and direct optical bandgaps of 1.12 and 1.55 eV, respectively.

**Figure 5:**
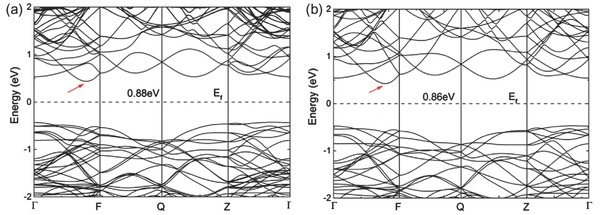
The band structure of SnSe is shown a) with SOC and b) without SOC. Reproduced with permission.^23^ Copyright 2015, Elsevier.

For bulk materials, the spin–orbit coupling (SOC) effect should also be considered. The Seebeck coefficient can be solved from the derivatives of the electronic density of states at the Fermi surface and the band velocity. The calculated band structure of SnSe along commonly explored high symmetry lines is presented below (**Figure** **5**).

The indirect bandgap is 0.86 eV, where the valence band maximum locates at Γ‐point and the conduction band minimum locates between Γ‐point and F‐point. By considering the SOC, the bandgap is 0.88 eV, this small change indicates the effect of SOC on the bandgap.^23^

## 3 Preparation Methods

For preparation of massive single‐crystalline SnSe bulk materials, melt‐growth method is usually employed. In the melt‐growth approach, 99.999% pure constituent elements of tin and selenium are mixed in stoichiometric ratio in a sealed quartz ampoule which is then kept in close vacuum (10^−5^ Torr). The ampoule is then slowly heated to 875 ± 2 °C and the mixture is left for 20 h after that. It is then shaken several times to homogenize the bulk material.^30^ However, low‐dimensional SnSe material, which promises superior properties than SnSe bulk crystals in many aspects, cannot be achieved by the bulk growth method. A comprehensive review on the preparation of microcrystals (it is characterized with several µm diameters), thin films, and nanostructures of SnSe by various technologies is given below.

The preparation of SnSe thin films on different substrates has been carried out by vapor phase methods, including physical vapor deposition (PVD) and chemical vapor deposition (CVD). Solution phase methods were the most popular approaches to produce SnSe microcrystals and low‐dimensional SnSe materials, though there are some reports that SnSe microcrystals and nanocolumns can also be prepared by PVD method. Other unique methods, such as high‐energy milling and ultrasonic spray pyrolysis technique, have also been employed to prepare powder‐like SnSe microcrystals and to deposit SnSe thin films,^31^ respectively. The brief descriptions of major characteristics, general conditions, and typical synthesis methods are given in the following section.

### 3.1 Solution‐Phase Methods

SnSe nanomaterials prepared by liquid phase methods are notable for their versatile morphologies. The shape and size of obtained SnSe nanostructures are controllable in liquid phase methods, which is satisfactory because some useful physical properties are closely related to shape of nanomaterials. The majority of reactants such as some organometallic precursors, organic solvent, and aqueous solution are toxic, sensitive to air, and expensive for vapor growth process. By contrast, there are green and facile liquid phase methods for preparing SnSe. In the following sections, synthesis methods are sorted by the reaction conditions and features.

#### 3.1.1 Solvothermal Method

**Figure 6:**
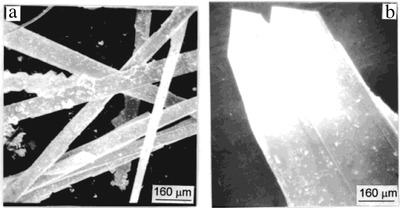
SEM image of SnSe single crystal prepared a) by route I and b) by route II. Reproduced with permission.^34^ Copyright 2000, American Chemical Society.

It is a classical method to synthesize SnSe_2_ which is a suitable and important precursor to obtain SnSe through heating at 400–500 °C.^32^, ^33^ The process of stoichiometric SnSe bulk synthesis requires the reaction of selenium (99.95%) and SnCl_2_ (99.95%) in a teflon‐lined autoclave at 180 °C for 7 d, two kinds of morphologies can be obtained (see scanning electron microscope (SEM) images in **Figure** **6**, 34) and the specific procedures are described in ref. 34. According to the report,^34^ morphology of rod like light‐gray crystals with ≈0.8–1.5 cm in length and ≈60–100 µm in radius or the plate like SnSe single crystal with the dimensions of 1200 µm × 300 µm and a thickness of 10 µm can be obtained.

#### 3.1.2 Hot Injection Method

Hot injection method is a chemical synthetic method of nanocrystal (NC) synthesis, in which the cold solution of one precursor is injected into hot solution of other precursor in the presence of a surfactant.^35^ Temperature of the hot solution is less than 100 °C in general, but in most conditions the temperature can be more than 100 °C.

In the phase‐control synthesis of SnSe nanocrystals, di‐tert‐butyl selenide was injected into a solution of anhydrous SnCl_2_, dodecylamine, and dodecanethiol (2.50 and 0.50 mL, respectively) at 95 °C.^28^ Afterwards, a dark‐brown solid was obtained. Change in phase between SnSe and SnSe_2_ revealed that adding a stoichiometric amount of di‐tert‐butyl selenide equals to that of anhydrous SnCl_2_ yield SnSe phase but a double amount of diselenide yield SnSe_2_ phase.

An interfacial synthesis method of colloidal SnSe quantum dots using hot injection was realized with nontoxic Se precursor under relatively mild temperature^36^ glycerol (30 mL) added to solution of stannous octoate (1.5 mmol) and oleylamine (6 mL) dissolved in toluene (30 mL) to form an interface. Cold fresh solution of sodium hydrogen selenide (NaHSe) was injected into the interface. The reaction lasted for 8 h and then SnSe quantum dots were obtained.

**Figure 7:**
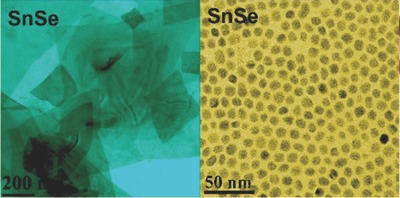
TEM image of SnSe nanosheets and quantum dots. Reproduced with permission.^37^ Copyright 2014, American Chemical Society.

In the typical synthesis of colloidal SnSe nanosheets using injection method, the Se precursor was TOP (trioctylphosphine)‐Se or tri‐tert‐butylphosphine‐Se, a toxic organometal substance, which was injected into solution of SnCl_2_ powder mixed with oleylamine, oleic acid, and dodecylamine. But in the modified nontoxic method, TOP‐Se was substituted for the oleic acid‐Se solution prepared by the reaction of Se powder (1 mmol) dissolved in oleic acid (10 mL). Details of the experiment are described in ref. 37. The SnSe NCs were synthesized by nontoxic way with a mean diameter of 7.5 nm (see transmission electron microscope (TEM) images in **Figure** **7**, 37).

The cubic nanoparticles of SnSe, which is designated as a new nanometric cubic phase of tin selenide, can also be synthesized by the hot injection method.^14^

#### 3.1.3 One‐Pot Method

In this method, all reactants including powders, solutions, and solvents are mixed at room temperature. Afterwards, the mixture is heated to initiate reaction. This process is actually the origin for the title of this technique, which makes the method facile to launch.^35^ Owing to the extremely poor solubility of selenium powder, some compounds containing selenium element can be utilized as Se precursor in one pot method.^29^

**Figure 8:**
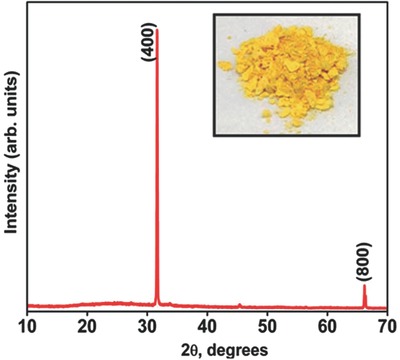
XRD spectra for SnSe nanosheets. Inset: yellow powder obtained. Reproduced with permission.^38^ Copyright 2015, Royal Society of Chemistry.

Colloidal SnSe nanosheets are synthesized by an innovative method, where bovine serum albumin (BSA) is added as a reducing agent, a stabilizing agent, and a shape directing template (see XRD spectra in **Figure** **8**). SnCl_2_ solution is used as Sn precursor and sodium selenosulphate (Na_2_SeSO_3_) is used as Se precursor. Equimolar concentrations of SnCl_2_ and Na_2_SeSO_3_ are mixed and BSA (1 mmol) is added with continuous stirring to form a greenish‐yellow solution. Finally the yellow powder is obtained by the evaporation of solvent.

**Figure 9:**
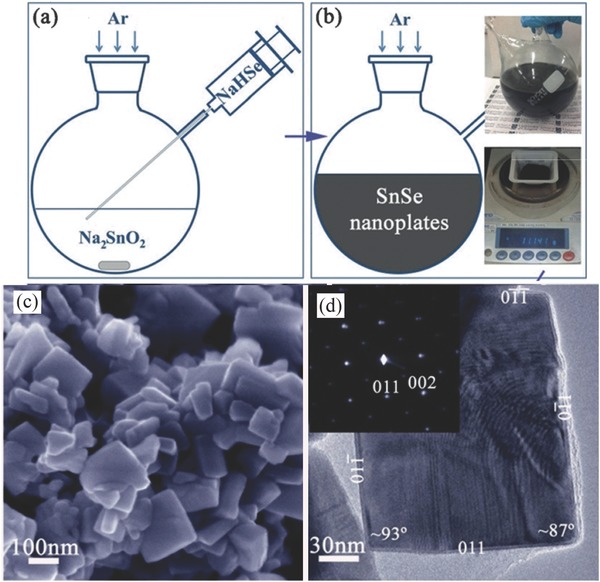
a,b) The schematic diagram of synthesis process. c,d) The SEM and TEM images. Reproduced with permission.^39^ Copyright 2016, Wiley‐VCH.

#### 3.1.4 Facile Surfactant‐Free Synthesis

This method is adopted due to surfactant‐free synthesis of material in which the mixture of NaHSe and Na_2_SnO_2_ solution is boiled for 2 h and the product can be hot pressed into pellets to obtain excellent properties.^39^ The thermoelectric power factors of SnSe nanoplates synthesized by this way were 80% higher than those of the equivalent materials prepared in the presence of a surfactant. On the other hand, the surfactant‐free synthesis can get high yields, i.e., SnSe nanomaterials in gram quantities, whereas the methods adopting organic surfactants or solvents have very less yields, i.e., in milligram quantities of materials.

The reaction can be carried out by injecting NaHSe solution (50 mL) into the Na_2_SnO_2_ solution, which leads to an immediate precipitation of SnSe nanoparticles. The reaction mechanism is given below

(1) $$\mathrm{NaHSe}+{\mathrm{Na}}_{2}{\mathrm{SnO}}_{2}+H_{2}O\to \mathrm{SnSe}+3\mathrm{NaOH}$$

Afterwards, the suspension is boiled for 2 h and then cooled down to obtain the phase‐pure nanoplates of orthorhombic SnSe. The SnSe sample was finally dried at 50 °C for 12 h. The SEM, TEM images and synthesis process are presented in **Figure** .^39^

#### 3.1.5 Hydrothermal Intercalation and Exfoliation Synthesis

**Figure 10:**
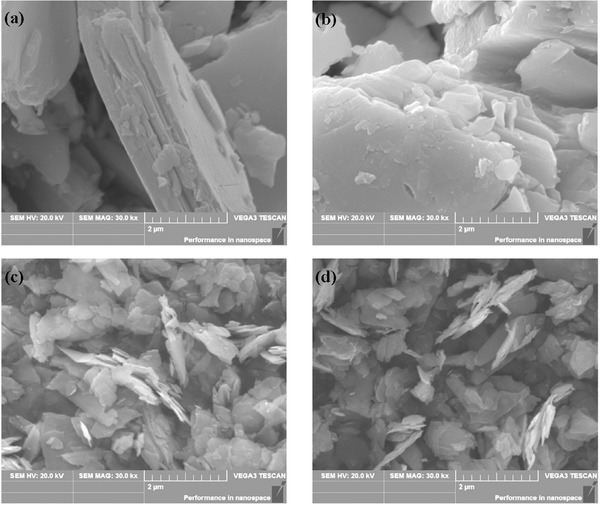
The SEM images of a,b) bulk SnSe; c,d) dispersed SnSe nanosheets. Reproduced with permission.^40^ Copyright 2016, Elsevier.

In this process, a practical Li‐intercalation hydrothermal exfoliation approach is introduced to synthesize step‐like SnSe nanomaterial.^40^ The primary principle of this method is hydrothermal intercalation and exfoliation, which is executed by the intercalation of Li ions into the bulk SnSe followed by the mixture in water to exfoliate the thin layer of SnSe material. The images of bulk SnSe and step‐like dispersed SnSe nanoplates are shown in **Figure** **10**. The rate of this process is very rapid due to the reaction between Li^+^ and water molecules just like atomic‐scale bombs, which let SnSe bulk separated into large quantity of monolayers or multilayers of SnSe NSs.

In this technique, SnSe bulk powder (0.33 g) is added into LiOH (0.64 g) dissolved in ethylene glycol (30 mL) with constant stirring. After that, the solution is transferred into a teflon‐lined auto clave (50 mL) and is maintained at 200 °C for 24 h. The most important step where extra care should be taken is while inserting the lithium ions into the interlayers of bulk SnSe, the final step is exposing the solid products in deionized water to obtain the SnSe nanosheets suspension.

#### 3.1.6 Sequential Deposition Method

SnSe‐CUB thin films can be obtained on SnS‐CUB thin film basic layer through the solution‐phase sequential deposition method.^16^ Synthesis of SnS‐CUB thin film basic layer on Corning microscope glass substrates treated in a dilute Na_2_S solution is the significant procedure to deposit SnSe thin film stacks of cubic phase, which is explained in ref. 16 in detail. The thickness of SnS‐CUB film deposited by this technique is in nanometer regime.

Subsequently, SnSe‐CUB thin film is deposited by the substrate coated SnS‐CUB inside the bath composition for SnSe. To obtain solution of Na_2_SeSO_3_, Se powder (2 g) and Na_2_SO_3_ (12.3 g) are refluxed in deionized water. For deposition bath solution, SnCl_2_ (0.7 g) is dissolved in acetone (5 mL), in which 3.5 m triethanolamine (CH_2_CH_2_OH)_3_N) (35 mL), 2.0 m solution of sodium hydroxide (NaOH) (18 mL), 0.5% polyvinyl pyrrolidone (0.25 mL), 0.2 m sodium selenosulfate (Na_2_SeSO_3_) solution (4.0 mL), and water (16 mL) are added and stirred. The bath is maintained at 17 °C for 1.5 h, followed by the same way for the second deposition during 1.5 h at 17 °C, which results in SnSe‐CUB thin film of about 200 nm in thickness.

A similar experiment with Na_2_S treated substrate without SnS‐CUB basic thin film deposited in SnSe bath twice at 17 °C resulted orthorhombic structure SnSe thin film of about 200 nm thickness.^15^

### 3.2 Vapor‐Phase Methods

#### 3.2.1 PVD of SnSe

PVD, involving no chemical reaction, is widely used in the synthesis of thin films. The source materials are gasified by physical method and then through the vapor phase process thin film is obtained on the substrate. The background pressure during PVD growth of thin films should be kept low enough to ensure effective mass transport from sources to substrates. While in some cases, for triggering and/or further manipulating the mass transport process, diluted inert gases can be employed. PVD technique can be classified into various types on the basis of heating technique used, i.e., resistive heating, flash evaporation, electron beam heating, laser heating, arc evaporation, sputtering, etc.

*Vacuum Evaporation (VE)*: In the process of VE, the source materials are transformed into vapors by some heating process such as ohmic heating, electron beam heating, high frequency induction heating, arc heating, or laser heating. The mixture of vapor is then transported toward the substrate surface where the molecules are then adsorbed on it. Some adsorbed molecules condense and initiate nucleation process and start the growth of crystalline thin film. A variety of VE techniques have been developed to attain thin films with high crystalline quality and good properties. Typical VE techniques for preparing Sn—Se compound thin films are briefly discussed below.

*Thermal Evaporation*: Bulk SnSe powder can be used as the starting precursor for the synthesis of SnSe thin film by thermal evaporation. The SnSe thin film is obtained on the substrate during the process of thermal evaporation. The widely used substrates for growth are glass, mica sheets, and sapphire.

**Figure 11:**
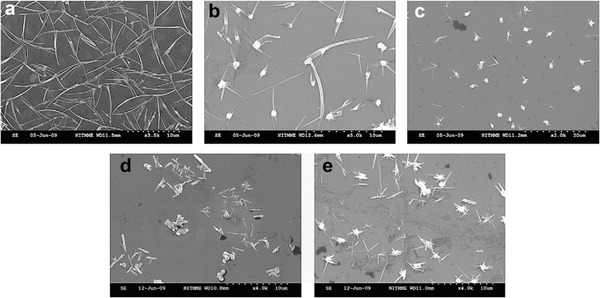
SEM images of SnSe thin films prepared at various substrate temperatures: a) room temperatures; b) 150 °C; c) 250 °C; d) 350 °C; e) 450 °C. Reproduced with permission.^41^ Copyright 2010, Elsevier.

Since the quality of the substrates also plays an important role in the thin film properties, the substrate cleaning is necessary to remove the impurities from the surface, i.e., oxides. First, the substrate is rinsed with hydrogen peroxide then with the vapors of acetone, trichloroethylene, and methanol, respectively, for several cycles. A Hind High Vacuum Coating Unit (Model 12‐A4D) is employed for the deposition of SnSe thin film which is a versatile laboratory model coating unit for thin film applications. This unit provides researchers with optional accessories like substrate heating, rotary drive, flash evaporation, electron beam gun evaporation. The thin film can be deposited on substrate at different temperatures such as room temperature, 150, 250, 350, and 450 °C. Molybdenum boat is applied to evaporate the precursor material and is heated by a current of 5 A. The evaporation lasts for 1 min in a high vacuum environment at a pressure of 5.5 × 10^−5^ Torr. The distance between the substrate and the source plays an important role during the deposition process. If the substrate is kept far away from the source, the smoothness of the film could be lost, however, if the substrate is too close to the source, its temperature would be affected by the source boat. An optimum distance of 14 cm is identified after several trials.^41^ In a reactive evaporation procedure to synthesize SnSe thin films, glass substrates are employed and an optimized substrate temperature of 523 ± 5 K at a base pressure of 10^−5^ mbar is reported for synthesizing crystalline SnSe thin films. XRD studies showed that the deposited thin films are orthorhombic whose orientation is parallel to [100] plane. The direct bandgap lies in the range of 1.2–1.4 eV and the indirect bandgap lies in the range of 0.6–1.2 eV. **Figure** **11** shows the surface morphologies of the prepared thin films. With the increase in the substrate temperature the surface morphology changes a lot as can be observed in Figure 11.^41^

*Inert Gas Condensation (IGC)*: IGC is a typical physical method for the synthesis of nanomaterials. Compared with other chemical synthesis methods, the nanostructure film produced by IGC is free from contamination. It has been shown that single‐crystalline layered‐compound thin films can be grown by the IGC method.^42^ For the deposition of SnSe thin film by IGC, SnSe powder is loaded in molybdenum boat and the thin film is deposited on clean glass substrate at 27 °C. The substrates are placed at distance of 13 cm from the source in order to obtain highly uniform film. The flow of Ar gas from the SnSe powder is kept at the pressure of 2 × 10^−3^ Torr. The deposited film thickness is dependent on the deposition time. The deposition conditions such as the flow rate of the inert gas, the distance between the source of evaporated materials and the substrate, the rate of deposition, and the substrate temperature are tuned to adjust the grain size. TEM images indicate that there is a direct relation between the film thickness and grain size, with the thickness increasing from 15 to 70 nm the relative increase in the grain size is reported as from 2 to 5.7 nm.^43^

**Figure 12:**
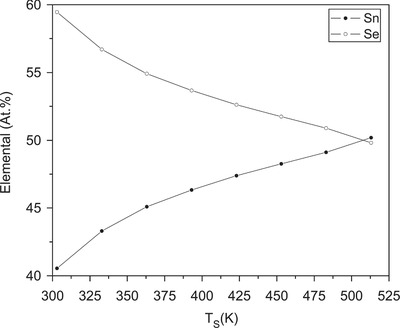
Dependence of elemental atomic percentages of Sn, Se in 300 nm thick films formed at different substrate temperatures. Reproduced with permission.^44^ Copyright 2007, Elsevier.

**Figure 13:**
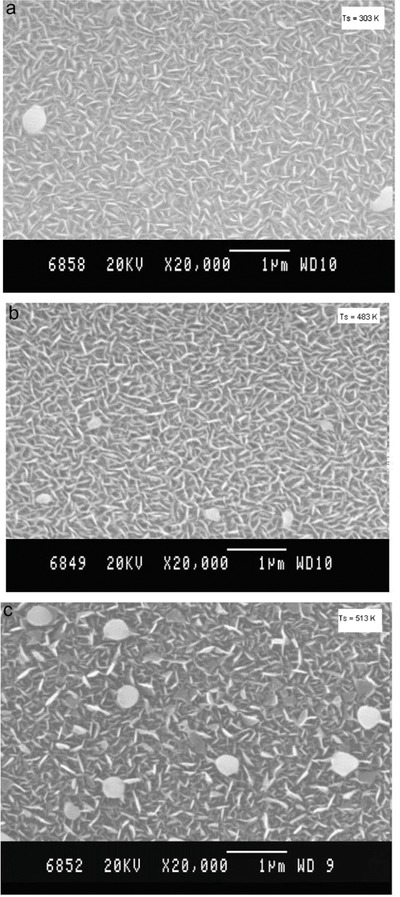
Scanning electron microscopy image of SnSe thin film (300 nm thick) deposited at substrate temperatures of a) 303, b) 483, and c) 513 K. Reproduced with permission.^44^ Copyright 2007, Elsevier.

*Flash Evaporation*: Flash evaporation is a method by which the source material is dropped onto the heating source in a small quantity and the powdered particles evaporate in a moment and form the film rapidly. In the process of flash evaporation, the SnSe powder can be served as the source material. A controllable vibratory spiral feeder is used to drop the powder on a molybdenum boat heated to 1423 K that causes the flash evaporation. The thickness of the obtained thin film which is monitored by a quartz crystal thickness monitor varied from 150 to 300 nm. Here, the other evaporation conditions such as the substrate temperature, the pressure, the evaporation rate are maintained at 303–513 K, 4 × 10^−6^ Torr, and 2 nm s^−1^, respectively. The lattice parameters of the deposited stoichiometric SnSe thin films at 513 K are reported as *a* = 11.50 Å, *b* = 4.15 Å, and *c* = 4.43 Å.^44^ The proportion of the Sn and Se content are highly dependent on the substrate temperature (**Figure** **12**). **Figure** **13** shows the SEM images of the SnSe thin films deposited at different substrate temperatures (white sheet‐like patterns in Figure 13a–c^44^). The grain size of the films increases with the increase of the substrate temperature as shown in Figure 13.

*Hot Wall Epitaxy (HWE)*: HWE is a kind of PVD technology which can grow high quality thin films. A typical HWE setup consists of a growth system, a heating and control system, and a vacuum system. The source material, the wall, and the substrate are all heated but the source temperature is higher than that of substrate temperature and is equal to that of the wall. Here, SnSe powder is taken as the source for the preparation of SnSe films. The chamber is baked at 473 K and pumped to vacuum before the deposition on the glass slides substrate. In a reported experiment the temperatures of the source and the wall were kept at 925 and 825 K, respectively. The substrate was heated by a radiant heater and the final temperature was varied from 373 to 573 K. SnSe films obtained at different substrate temperatures were of different thicknesses of 0.5–8 µm.^45^

**Figure 14:**
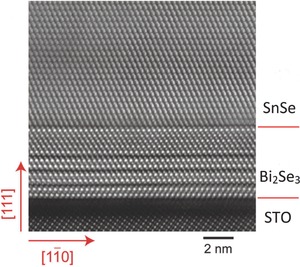
Cross‐section HRTEM image of a 16 nm SnSe film grown on 5 quintuple‐layer (QL) Bi_2_Se_3_ film. STO refers to SrTiO_3_. Reproduced with permission.^51^ Copyright 2015, Wiley‐VCH.

*Molecular Beam Epitaxy (MBE)*: MBE is also based on the evaporation of sources. It has been successfully utilized to synthesize layered‐materials^46^, ^47^ and quantum structures.^48^, ^49^, ^50^ If the material to be deposited on the substrate is the same material as the substrate then process can be called as homoepitaxy, otherwise if both are different then it is called as heteroepitaxy. The structure and orientation of the thin film are related to those of the substrate. In the ultrahigh vacuum environment, the constituent element molecules are sprayed on the surface of the substrate and form thin film at corresponding substrate temperature. MBE growth of SnSe films has been realized on the Bi_2_Se_3_ surface. Before the preparation of SnSe, Bi_2_Se_3_ was grown first so that it could serve as the substrate. In the synthesis of SnSe, the flux ratios of Sn and Se were kept stoichiometric. **Figure** **14** shows the high‐resolution transmission electron microscopy (HRTEM) image of 16 nm thick SnSe film, grown on a 5 nm thick Bi_2_Se_3_ film.^51^

*Sputtering*: In this technique, target material and substrate serve as cathode and anode, respectively. The space between the thermionic cathode and the auxiliary anode is filled with argon and forms the plasma arc column of low voltage and large current. Electrons are collided with the argon atoms, which lead to its ionization and produce a large quantity of ions. In the process of synthesizing SnSe nanosheets, this technique is called plasma arc beam sputtering. Tungsten is used as the cathode, 99% pure Sn and Se powders mixed with a stoichiometric ratio are pressed into ingot and serve as anode. The pressure of Ar gas is kept at around 75 Torr. When the direct current arc discharge is kindled, a large current of 100 A and a low voltage of 15 V is established. Such technique was employed in a report where the discharge process lasted for 5 min and SnSe nanosheet of about 25 nm was obtained at the water‐cooling wall surface.^52^

#### 3.2.2 CVD of SnSe

CVD is a technique often exploited to synthesize thin films and nanostructured materials. Typical CVD reactions include thermal decomposition reaction, building‐up reaction, oxidation–reduction reaction, single displacement reaction, etc. The appliance of CVD must meet three conditions: (1) The reactant gas should generate a sufficient vapor pressure and be introduced into the reaction chamber at an appropriate speed and a deposition temperature. (2) The reaction product of solid film material except SnSe must be volatile. (3) Deposited films and the substrate material must have a sufficiently low vapor pressure. The product quality is influenced by the deposition temperature, the pressure in the deposition chamber, and the proportion of reactant gas. The CVD technique comprises atmospheric pressure CVD (APCVD), low pressure CVD, plasma enhanced CVD, vapor phase epitaxy, metalorganic CVD, and atomic layer epitaxy.

*APCVD*: APCVD conducts chemical vapor deposition at atmospheric pressure. It is an original method with a simple reaction system. Despite the poor homogeneity and ability to cover the terrace, APCVD is efficient at obtaining products. The deposition of SnSe can be done on SiO_2_ coated bottom and top substrates in a 99% pure dinitrogen atmosphere. SnCl_4_ and diethyl selenide are reported to be used as reactant gases and placed into two different bubblers. The two gases were carried by hot nitrogen and mixed in the reaction chamber. The reaction was carried out at 400–650 °C and lasted for 1 min. Since the bottom substrate was directly heated and the top was not, there existed a temperature difference of 75–100 °C that led to the different properties of the films obtained on different substrates.^53^

*Atomic Layer Deposition (ALD) of SnSe*: Atomic layer deposition, a technique that is similar to CVD, is used to deposit the monatomic film on the substrate layer by layer. During the deposition process the chemical reaction of the new monatomic film is directly related to the prior layer that leads to the deposition of single atomic layer during each reaction. The vapor precursor pulses are passed into the reactor alternately to launch reactions and the product is adsorbed on the substrate. In this process Et_4_Sn and H_2_Se are used as precursors. The growth is carried out in a flow type vacuum facility which contains a chamber, a system of impulse inlets of the precursor vapors and permanent evacuation of the by‐products of the reaction, heater of the substrate, a trap for the products of the reactions, and a system for control and monitoring of the process with the aid of a personal computer. The interaction is performed after successive vapor precursor pulses are passed in. The deposition temperature is controlled between 200 and 450 °C. The vapor pressure values are 0.4 and 1.0 Torr for Et_4_Sn and H_2_Se, respectively. In a typical cycle the interval time for the gas purging is 1.0 s and the inlet time for the precursors is 0.2 s. In a reported work, SnSe samples were synthesized by conducting 1000 ALD cycles. It was found that the more the cycles conducted, the thicker the samples would be.^54^

## 4 Electrical and Thermoelectric Properties

In this section, we first discuss the properties of pure bulk SnSe single crystals and polycrystals. Then, methods to enhance their *ZT* values will be discussed.

The dimensionless figure of merit (*ZT*) is defined as

(2) $$ZT={\left(S\right)}^{2}\sigma T/\left(k_{e}+\;k_{l}\right)$$

(*S*), σ, *k*_e_, and *k*_l_ stand for the Seebeck coefficient, electrical conductivity, electronic thermal conductivity, and lattice thermal conductivity, respectively. *ZT* determines the efficiency of thermoelectric materials. Here, the power factor, which is often used to measure the thermoelectric property, is defined as

(3) $$\mathrm{PF}={\left(S\right)}^{2}\sigma$$

### 4.1 SnSe Single Crystals

It is necessary to find materials with inherent good TE properties because traditional methods to improve TE properties are too complex and costly. SnSe is a material with intrinsic ultralow lattice thermal conductivity. The highest *ZT* values of 2.6 along the *b*‐axis and 2.3 along the *c*‐axis have been observed at 923 K. However, the *ZT* value along the *a*‐axis is small and is measured to be 0.8 due to the poor electrical conductivity.^8^

**Figure 15:**
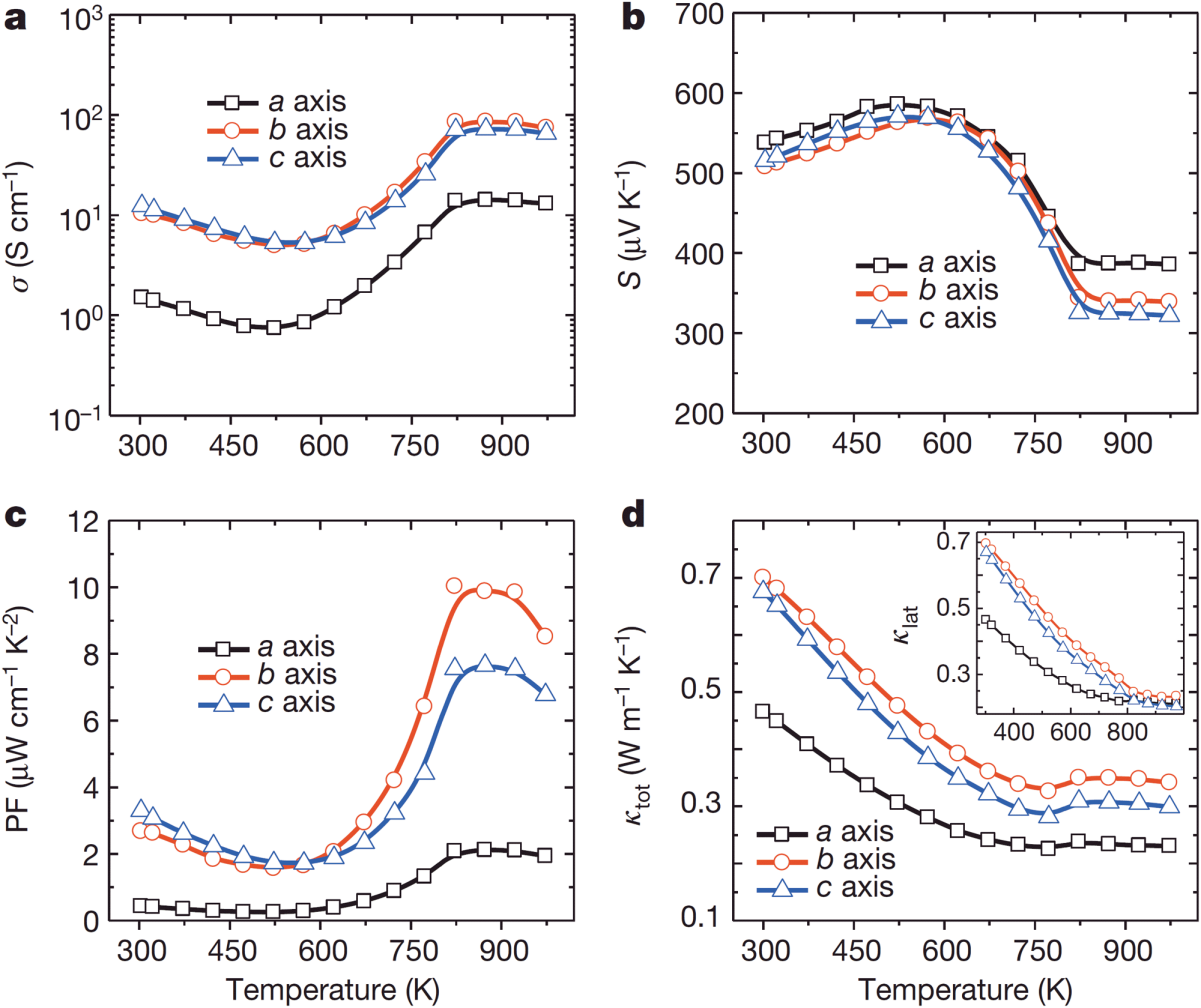
Thermoelectric properties as a function of temperature for SnSe crystals. a) Electrical conductivity. b) Seebeck coefficient. c) Power factor, PF. d) Total thermal conductivity, *k*_tot_. Inset: lattice thermal conductivity, *k*_lat_ (same units as *k*_tot_), versus temperature (same units as main panel). Reproduced with permission.^8^ Copyright 2014, Nature Publishing Group.

**Figure 16:**
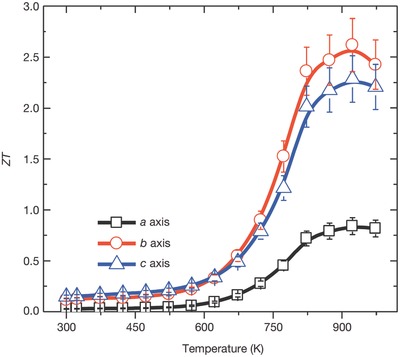
*ZT* values along different axial directions; the *ZT* measurement uncertainty is about 15% (error bars). *ZT* values are shown on the blue, red, and grey arrows; colors represent specimens oriented in different directions. Reproduced with permission.^8^ Copyright 2014, Nature Publishing Group.

Along different axes, the electrical conductivity (σ) and thermal conductivity (κ) both exhibit huge difference while their temperature (*T*)‐dependent trends are analogical. The σ–*T*, κ–*T* and (*S*)–*T* curves ((*S*) refers to Seebeck coefficient) can all be roughly divided into three sections in different temperature ranges (see **Figure** **15**).^8^ The first section, ranging from 300 to 525 K, exhibits a metallic transport behavior: (*S*) increases and σ decreases as temperature rises; the second section, ranging from 525 to 800 K, shows a thermally activated semiconducting behavior: (*S*) decreases and σ increases as temperature rises. The upturn at 525 K is due to the thermal excitation of carriers. The thermal conductivity κ exhibits a decreased trend in both sections. In the third section, where the temperature is above 800 K, all the parameters remain constant, which may be due to the phase transition from P_nma_ to the C_mcm_ space group. The *ZT* merit and absolute temperature are positively correlated before *T* reaches maximum points of 923 K. Three obviously different sections can also be observed in the *ZT*–*T* curves (see **Figure** **16**). In the first and the third sections, the *ZT* is nearly temperature‐independent because of the balance of σ, (*S*), and *k* while in the second section, the *ZT* value increases rapidly as *T* rises because of the combined effect of increased power factor (PF) and κ.^8^, ^55^

Compared to other advanced TE materials, SnSe is promising for its ultralow thermal conductivity and moderate power factor.^8^ According to Ding et al., SnSe shows much larger bandgap value of 0.69 eV than those of traditional thermoelectric materials, thus lower carrier concentration around the Fermi level is induced. This proper carrier concentration contributes to large PF.^56^ Ultralow thermal conductivity in SnSe is novel in consideration of the absence of complex improved methods such as nanostructuring and all‐scale hierarchical architecting. It attributes to the strong anharmonicity in bonding owing to its special structure.^8^

### 4.2 SnSe Polycrystals

**Figure 17:**
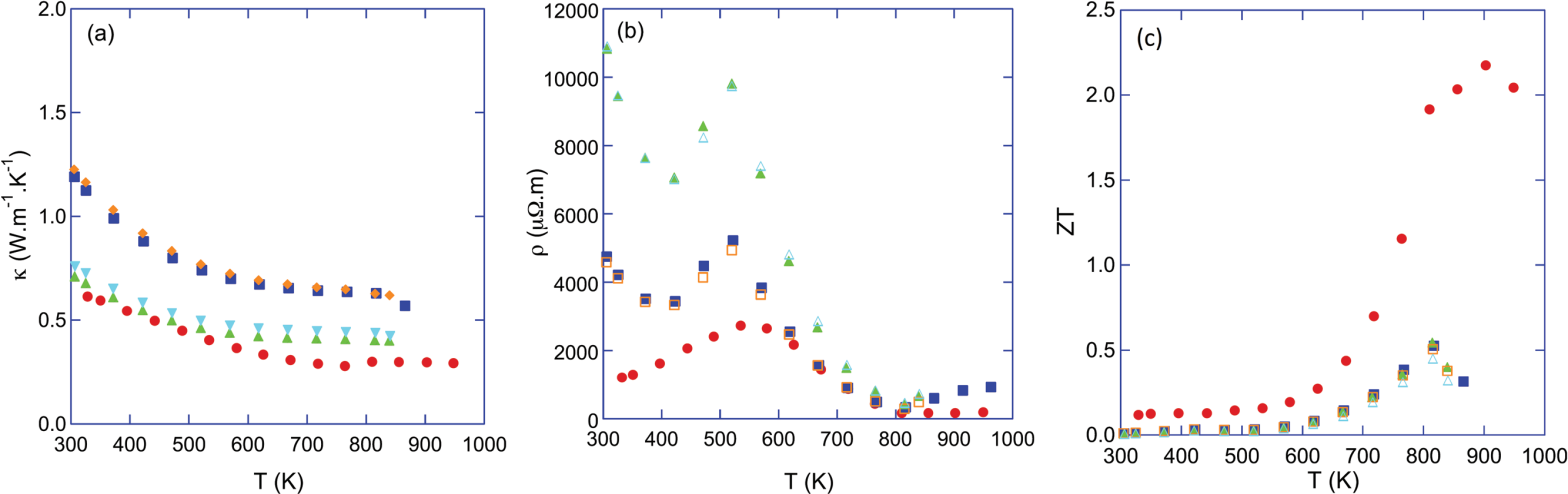
Temperature dependence of the electrical properties of SnSe measured in both directions: a) thermal conductivity κ, b) electrical resistivity ρ, and c) the dimensionless figure of merit *ZT*. Both properties were measured parallel (triangle symbols) and perpendicular (square symbols) to the pressing direction in two different samples. The mean value of the data collected on single crystals has been added for comparison (filled circle symbols). Reproduced with permission.^55^ Copyright 2014, AIP Publishing.

The commercial applications of single crystals are limited due to their fragility and carefully controlled conditions required for synthesis processes. Further research on polycrystalline SnSe became necessary. The extreme interface or surface scattering causes low κ and σ in polycrystalline materials while the absence of those in single crystalline materials induces high electrical and thermal conductivities,^57^ the *ZT* value of polycrystalline SnSe may be close to that of single crystalline SnSe. Unfortunately, experimental results have revealed that the *ZT* value which is lower than 0.6 in polycrystalline SnSe is even much lower than that in its perfect crystals. It is confusing that thermal conductivities in polycrystals are even higher than those in single crystals. The reason for the discrepancy between them still requires further research.^8^, ^55^, ^58^ Lenoir and co‐workers^55^ synthetized anisotropic p‐type SnSe polycrystals of which all parameters related to *ZT* in polycrystals are worse than those in single crystals (see related parameters in **Figure** **17**). Below the temperature where the phase transition occurs, (*S*) is only a little lower than that in single crystals while above that temperature, (*S*) in polycrystals decreases rapidly and (*S*) in single crystal attains a nearly constant value by contrast.^55^ It is the primary reason why the maximum point does not appear at the same temperature as single crystals. The overall temperature‐dependent trend of σ and *k* in polycrystals is similar to those in single crystals, nonetheless, σ is lower and κ is larger and the anomaly of σ around 530 K is more significant in polycrystals.^8^, ^55^, ^58^

It is highly noticed that during heating and cooling, SnSe polycrystals show hysteresis in σ and (*S*) due to some reversible processes, which is also observed in Ag‐doped and Tl‐doped polycrystals.^58^, ^59^

### 4.3 Doped SnSe

The properties of pure SnSe are excellent but there are several problems limiting its commercial applications. First, the efficiency of TE materials is actually determined by the average value of *ZT* over the temperature‐dependent *ZT* curves between hot and cold end temperatures.^60^ But the average *ZT* values of pure single crystals and polycrystals are both insufficient.^8^, ^55^, ^58^, ^61^ Second, polycrystals are more competitive in commercial applications in view of better mechanical properties and low costs. However, in comparison to single crystals, SnSe polycrystals exhibit poor TE properties.^8^, ^55^, ^58^ Therefore, state‐of‐the‐art method such as metal‐doping was used for further improvement. Doping in SnSe is a challenge due to its special structure.^11^ Fortunately, there are several effective dopants found in previous experiments: S, silver (Ag), sodium (Na), bismuth chloride (BiCl_3_), thallium (Tl), copper (Cu), zinc (Zn), and iodine. Sulphur and copper can help to reduce the thermal conductivity; Ag, Na, Zn, and Tl are adopted to tune carrier concentrations; and iodine and BiCl_3_ are utilized to turn intrinsic p‐type SnSe materials into n‐type ones which are predicted to have better thermal properties.^11^, ^58^, ^59^, ^61^, ^62^, ^63^, ^64^, ^65^

#### 4.3.1 Ag‐Doped and Na‐Doped SnSe

Ag and Na are used as dopants in SnSe materials owning to their effect on carrier concentration promotion and the Fermi level lowering.^11^, ^58^, ^61^ These two factors lead to lower (*S*) but higher σ. The former is of benefit to *ZT* improvement while the latter is not. Thus, PF may increase if the lower (*S*) can counteract the effect of large σ. Na has a better performance than Ag, judging by higher power factor and lower κ in Na‐doped bulk materials. Different electronic transport properties are induced by different valence states and effects on electron scattering of Ag and Na. Moreover, Na‐doped SnSe exhibits better thermal transport properties attributed to significant difference in mass and size between Sn and Na atoms, which causes prominent scattering of phonons and thus magnifies κ.^61^ However, Na‐doped polycrystals exhibit instability upon repeated heating and cooling.^58^ Consequently, Na dopant is only applied in single crystals.^11^, ^61^

For polycrystals, Ag dopant increases the maximum *ZT* value slightly to 0.6 at 750 K. X‐ray diffraction and back‐scattered electron displays the existence of AgSnSe_2_ with cubic structure. This secondary phase is isolated and results in increased volume fraction that leads to poor thermal transport properties. This together with the lower mobility is the primary reason why continuous increase in Ag content fails to further increase *ZT*. Only the sample with 1% Ag content was observed to have the maximum *ZT*.^58^

For single crystals, the experimental result of Na‐doped SnSe is satisfactory. Along the crystallographic *b*‐axis, the maximum *ZT* value is measured to be close to 2 at around 800 K, which is much lower than that of the undoped SnSe.^8^, ^11^, ^61^ Nevertheless, the promotion of TE performance below that temperature is significant. Peng^61^ achieved the average *ZT* value of 1.17 over the temperature range from 300 to 800 K but Zhao et al.^11^ observed 1.34 from 300 to 773 K. Upon comparing different doping concentrations, 3% impurity level is confirmed to be the best. The primary reason of this result is that in doped material σ is at least 50 times higher than that in undoped crystals. Because of its inferior electronic properties, Ag‐doped single crystals show unsatisfactory features of much lower *ZT* value even smaller than 1.0 in Sn_0.99_Ag_0.01_Se and Sn_0.98_Ag_0.02_Se samples over the temperature range from 300 to 800 K.^11^, ^61^

#### 4.3.2 S‐Doped SnSe

SnS shows some similarities to SnSe and exhibits novel TE properties.^56^ Accordingly, S was incorporated in SnSe materials hoping that it will improve *ZT* but the maximum *ZT* value after the incorporation of S was found to be 0.82 in SnS_0.2_Se_0.8_ sample at 823 K, which is primarily attributed to the reduced κ and secondly to the slightly increased (*S*). Hall carrier concentration and Hall carrier mobility of SnS_1‐_ *_x_*Se*_x_* show a decreasing trend with the Se content rising and thus cause the decreasing trend of (*S*) and increasing trend of σ. Although in SnS_1‐_ *_x_*Se*_x_* (0.2 ≤ *x* ≤ 0.8), κ increases with Se content diminishing, atomic disorder attributed to the random distribution of isoelectric atoms leads to lower κ than undoped SnSe.^10^, ^63^

#### 4.3.3 Iodine‐Doped SnSe

Zhang et al. first converted the electrical conductivity of SnSe to n‐type.^10^ Prior to this work, Chen et al. (cite thermoelectric properties of p‐type polycrystalline SnSe doped with Ag) found that 5% additional Sn can transform SnSe materials into slightly n‐type. However, the result is unstable: in some experiments, SnSe_0.95_ shows positive (*S*). Moreover, extra iodine can also incorporate electrons into SnSe. Accordingly, substituting iodine for Se atoms in SnSe is a feasible method for the electrical conductivity conversion of SnSe materials. Iodine‐doped SnSe shows an overall decrease trend in (*S*), which is different to the undoped SnSe. Since (*S*) in n‐type semiconductors is negative, (*S*)^2^ in iodine‐doped SnSe increases with temperature increasing ranging from 300 to 800 K, which leads to a fact that (*S*), σ, and κ reach highest values in the same temperature interval. SnSe_0.97_I_0.03_ sample got the best result and *ZT* of about 0.8 at about 773 K was obtained.^10^

#### 4.3.4 SnSe Doped with Both Iodine and S

As mentioned above, S doped SnSe has reduced κ and thus S has been incorporated into iodine‐doped SnSe polycrystals by Zhang et al.^10^ According to the Debye approximation, the lowest κ_l_ in SnSe_0.87_S_0.1_I_0.03_ is calculated to be about 0.26 W m^−1^ K^−1^ at about 770 K, which is much lower than that in undoped and I‐doped SnSe (κ_l_ of pure SnSe and SnSe_0.97_I_0.03_ at 770 K are about 0.4 and 0.37 W m^−1^ K^−1^, respectively) at 770 K. Consequently, a *ZT* value as high as about 1.0 was obtained at about 773 K.^10^

#### 4.3.5 BiCl_3_‐Doped SnSe

Although high *ZT* values have been obtained, carrier density of iodine‐doped polycrystals is small^10^ and then BiCl_3_ was doped in polycrystals for a larger carrier density in n‐type SnSe and this successfully increased the carrier concentration by two orders of magnitudes. The value of σ in BiCl_3_ also increased significantly which is attributed to the promotion of the carrier concentration and carrier mobility.^62^ Nevertheless, the maximum *ZT* of 0.7 at 793 K in the SnSe_0.95_‐BiCl_3_ system does not exceed that of the iodine doped SnSe.

#### 4.3.6 Tl‐Doped SnSe

Tl is also used for the carrier concentration tuning. Hole concentration in Tl doped polycrystals is one order of magnitude more than that of undoped polycrystals. Specifically, the peak electrical conductivity of Sn_0.99_Tl_0.01_Se exceeds 10^4^ Ω^−1^ m^−1^.The maximum *ZT* value reaches 0.6 at 725 K, which is close to that of Ag‐doped one. Strength and ductility improvements are achieved in Tl‐doped SnSe.^58^, ^59^

#### 4.3.7 Cu‐Doped SnSe

Singh et al.^65^ have fabricated SnSe polycrystal samples doped with 2% of aluminum (Al), lead (Pb), indium (In), and Cu individually. All of these samples except In‐doped ones showed greater TE properties than undoped polycrystals. Specifically, The maximum *ZT* of Al‐doped, Pb‐doped, and In‐doped SnSe are 0.6, 0.5, and 0.09, respectively. *ZT* of Cu‐doped SnSe attained the most satisfying value of 0.7 at 773 K, which is mainly due to the ultralow thermal conductivity attributed to the presence of Cu_2_Se second phase associated with intrinsic nanostructure formation of SnSe. In addition, lower cost than some other dopants such as Te and Ag makes Cu a more promising dopant to increase the thermoelectric figure of merit *ZT*.^65^

#### 4.3.8 Zn‐Doped SnSe

The best result in p‐type polycrystals was obtained by Li et al. incorporating Zn into SnSe. The maximum *ZT* attained by this dopant was 0.96 at 873 K for Zn_0.01_Sn_0.99_Se sample. The tremendous advancement is attributed to the high electrical conductivity and Seebeck coefficient.^64^ The worth mentioning thing here is that Li et al. obtained a better *ZT* of 0.63 at 873 K from pristine polycrystals than the result reported by Sassi et al.^55^, ^64^

### 4.4 Nanostructured SnSe

Researchers are facing one difficult problem that the increase in (*S*) often causes σ to decrease and the decrease in κ usually reduces σ at the same time.^66^, ^67^ Nanoscale phenomenon has confirmed this contradiction in many other TE materials.^67^ Zhang and co‐workers studied the single‐layered sheet theoretically and obtained good results. The predicted *ZT* value reached 3.27 along the armchair (zigzag) direction with optimal n‐type carrier concentration and is larger than that of the single crystal.^8^, ^68^ The enhancement benefits from higher power factor owing to the quantum‐confinement effects.^68^ However, experimental research in this direction is still insufficient. The weak van der Waals force and strong covalent bonds within each layer suggest the possibility to extract a single‐layered SnSe sheet from the layered bulk material.^68^ The next step now is to validate this theory through experiments.

Cubic phase SnSe reportedly recently exhibits desirable thermoelectric properties and a *ZT* value of (0.998) is obtained at ≈200 K which is higher than orthorhombic structure SnSe (polycrystalline and bulk). The narrow bandgap and phonon constraining effects make cubic SnSe a promising material in the thermoelectric application.^69^

## 5 Optoelectronic Properties

As a newborn thermoelectric material, SnSe needs further study. Meanwhile, SnSe initially got attention by the research community for its wonderful behavior in the field of photoelectricity.

### 5.1 The Bulk SnSe

#### 5.1.1 Bandgap

SnSe belongs to IV–VI group and has orthorhombic crystal system. SnSe is a narrow bandgap semiconductor material with good optoelectronic properties. The bulk SnSe is an indirect bandgap material. Its indirect bandgap of 0.89 eV is in great agreement with optical‐absorption measurement of 0.90 eV.^22^ The value of the direct bandgap is 1.30 eV.^70^ The values of the bandgaps are different in the SnSe thin films and nanostructures.

#### 5.1.2 Optical Absorption Rate

The energy conversion efficiency of any material is closely related to its bandgap because of the larger overlap between their absorbance and the solar spectrum. Films with a thickness value of 300 nm can absorb the major part of solar energy. The optical absorption coefficient (α) is about 10^5^ cm^−1^ in the visible region.^70^

#### 5.1.3 Electrical Properties

The bulk SnSe also exhibits the favorable electrical properties. The electrical conductivity of this p‐type material has a linear relationship with the relaxation‐time. When the relaxation‐time was assumed to be 10^−14^ s in the bulk SnSe, the electrical conductivity increased with the temperature and a constant value of 5 Ω^−1^ cm^−1^ in the room temperature was obtained.^56^

Compared with other photovoltaic materials, SnSe has the advantage that it is composed of abundant and nontoxic elements,^71^ which make SnSe a potential candidate as an absorber material for photovoltaic device.^70^

To further enhance the optoelectronic properties of SnSe, various techniques have been applied like fabricating thin films of SnSe, nanostructures, or doping it with impurity elements.

### 5.2 SnSe Thin Films

#### 5.2.1 Bandgap

Bandgap of the SnSe thin film is related to the synthesis method and conditions. Some bandgap values of orthorhombic SnSe thin films reported by other authors are recorded in **Table** **3**.^72^ In addition, a direct bandgap value of ≈1.4 eV is observed in SnSe‐CUB thin films.^15^

**Table 3:** SnSe bandgap energy (eV) reported by various workers (reproduced from ref. 72)

Sl. No.	Method of deposition	Bandgap energy	Nature of transition	Ref.
1.	Vacuum	1.26	Direct	[72]
2.	Vacuum	1.21	Direct	[73]
3.	Reactive evaporation	1.21	Direct	[74]
4.	Laser ablation	0.94	Direct	[75]
5.	Vacuum	0.935	Indirect	[76]
6.	Electrodeposition	1.30	Indirect	[77]

#### 5.2.2 Optical Absorption Coefficient

The optical absorption coefficient α can be calculated by the equation

(4) $$\alpha =\frac{1}{t}\ln\frac{1}{T_{x}}$$

where “*t*” is the thickness of the thin film, and *T*_x_ is the transmittance which represents the ratio of transmitted light intensity to incident light intensity.

The relation between the absorption coefficient with the photon energy *hv* is given as

(5) $$\alpha \left(hv\right)=A\left[hv-E_{g}\pm E_{p}\right]$$

where *E*_p_ and *E*_g_ refer to the phonon energy and the energy gap, respectively.

**Figure 18:**
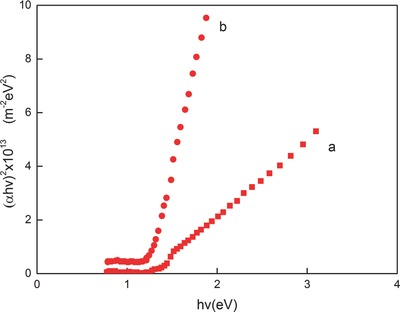
Plots of (*αhv*)^2^ versus *hv* for SnSe thin films a) as prepared and b) annealed at 300 °C. Reproduced with permission.^72^ Copyright 2000, Wiley.

The relation between the absorption coefficient and the photon energy is shown in **Figure** **18**. The absorption coefficient increases with the increase in photon energy.^72^

#### 5.2.3 Photoresponse

The optoelectronic properties of the SnSe thin films prepared by resistive evaporation at 373 K on glass substrates have been studied. The studies have shown that the optoelectronic properties have a close relationship with the light intensity and the temperature.

The photocurrent has a linear relationship with the light intensity. The growth rate of the curves decreases to a lower level when the intensity gets 20 mW cm^−2^. This indicates that the photosensitivity will decrease at higher light intensities.

The photocurrent is also influenced by the temperature, initially it increases with the increase in the temperature and later decreases with the continuous increase in temperature and attains a peak at 350 K. The decreasing is attributed to the fact that the traps filled by excitons at low temperature will be emptied by raising the temperature.

The change in the photoconductivity relates to the shallow and deep traps in the material. The photoconductivity increases and reaches the maximum value under the illumination. After the light is switched off, the photoconductivity decreases rapidly due to the recombination of excess electrons and holes.^78^ The vacuum deposited SnSe thin films also exhibit similar optoelectronic properties.

**Figure 19:**
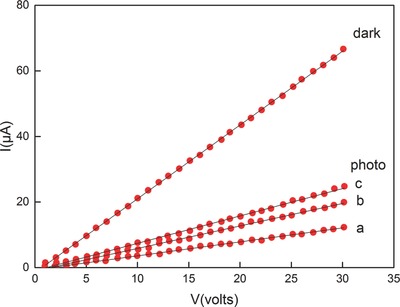
Dark current and photocurrent as a function of voltage at different levels of illumination (a: 600 lux, b: 1800 lux, c: 3000 lux) in SnSe thin films. The photocurrent is calculated by subtracting the dark current from the total current measured. Reproduced with permission.^72^ Copyright 2000, Wiley.

The dark current and photocurrent increase linearly with the increase in the voltage in the studied regions (0–30 V). The photocurrent is calculated by subtracting the dark current from the total current measured. The photocurrent also increases with the photoillumination level. In the light intensity of 3000 lux, the dark conductivity (σ_d_) and the change in electrical conductivity under illumination (σ_ph_ − σ_d_) are 5.56 × 10^−9^ and 1.94 × 10^−9^ Ω^−1^ m^−1^, respectively. When the intensities reach 600, 1800, and 3000 lux, the corresponding photosensitivity values [(σ_ph_ − σ_d_)/σ_d_] are 0.17, 0.27, and 0.35 (see **Figure** **19**, 72), respectively.

### 5.3 The SnSe Nanostructures

Compared with the SnSe bulk materials, the SnSe nanostructure has a large specific surface area and quantum confinement effects which will affect their optical and electronic properties. These properties lead to a tunable bandgap and high photosensitivity.^79^

#### 5.3.1 Bandgap

Structures and preparation methods of SnSe nanostructure affect its bandgap. Bandgap energy values reported by various authors for SnSe nanostructure are given in **Table** **4**.

**Table 4:** Bandgap (eV) energy values reported by various workers

No.	Method of deposition	Nanostructure	Bandgap energy		Ref.
			direct	indirect	
1.	Chemical synthesis	Nanocrystals	1.74	1.2	80
2.	One‐pot colloidal	Nanosheets	1.10	0.86	27
3.	One‐pot colloidal	Nanoflowers	1.05	0.95	27
4.	CVD	Nanowires	1.03	0.92	101

Unlike SnSe bulk materials, blueshifts in the SnSe nanocrystals are observed because of the quantum confinement effects in the nanostructure.^80^ The bandgap increases with the decrease in grain size.^81^ In other words, it is possible to customize bandgap by controlling the grain size of material.

#### 5.3.2 Photoresponse

**Figure 20:**
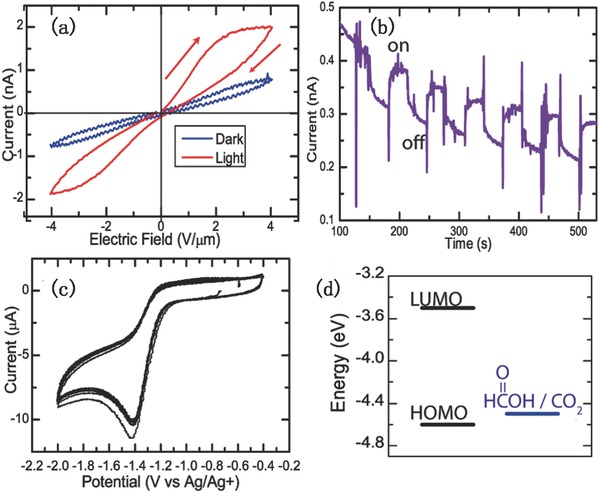
Current–voltage characteristics of formic acid treated SnSe NC films. a) *I*–*V* curves, b) transient photocurrent observed at a bias of 2 V µm^−1^ under 100 mW cm^−2^ illumination turned on and off at 45 s intervals. c) Cyclic voltammogram of oleic acid capped SnSe NCs using a Ag/Ag+ reference electrode and d) corresponding HOMO–LUMO energy levels. Reproduced with permission.^81^ Copyright 2010, American Chemical Society.

The photoresponse of the SnSe nanocrystals has also been studied. **Figure** **20** shows *I*–*V* hysteresis loops emerging from charge trapping in surface states or photo‐electrochemical interactions between the nanocrystal and surface bound ligands (see Figure 20, 81).

## 6 Applications and Perspectives

### 6.1 Photovoltaic Application

Based on favorable optoelectronic properties, SnSe possesses good potential in the application of solar cells. Open circuit voltage, short‐circuit current, fill factor, and power conversion efficiency are the main parameters to evaluate the performance of the solar cell.

An huge study can be found on the applications of SnSe materials in solar cells since 1990 and a detailed review about solar cells based on orthorhombic SnSe thin films has also been published in 2016.^82^ Orthorhombic SnSe thin films reported in 2014 deposited on n‐Si single crystal showed good optoelectronic properties. When the cell was connected to a load resistance under a halogen lamp light illumination with an input power density of 50 mW cm^−2^ at room temperature, an open circuit voltage of 425 mV, a short‐circuit current density of 17.23 mA cm^−2^, and a power conversion efficiency of 6.44% were observed.^82^

SnSe‐CUB is considered as a novel solar cell absorber for its desirable optical bandgap. A light generated current density of 23 mA cm^−2^ and a maximum of 29 mA cm^−2^ were predicted in a thin film of 200 nm in thickness of SnSe‐CUB in a solar cell.^15^ SnS‐CUB/SnSe‐CUB stacks, built by chemical deposition, are promising for the photovoltaic (PV) applications attributing to a more suitable optical bandgap value determined by the solid solution and also has higher electrical conductivity than the SnSe‐CUB thin films. A light generated current density of 28–31 mA cm^−2^ in solar cells for air mass 1.5 global solar radiation can also be predicted.^16^

The measured values were not in agreement with the theoretical values (theoretical value: an open circuit voltage of 744 mV, a short‐circuit current density of 43 mA cm^−2^, and power conversion efficiency of 32%) indicating the demand of further improvement on SnSe‐based solar cells.^8^

Compared with other conventional absorber layers, including CuIn*_x_*Ga_1‐_ *_x_*Se_2_ (CIGSe), CuInSe_2_ (CISe), and Cu_2_ZnSnSe_4_ (CZTSe), the efficiency of SnSe‐based solar cell is much lower. Moreover, there are difficulties limiting the performance of the SnSe‐based solar cells, including bulk defects and secondary phase formation in SnSe, band alignment and density of defect states at SnSe/n‐buffer layer interface, and nature of back contact.

The conversion efficiency of solar cell is influenced by different parameters like the growth condition, crystalline quality, p–n junction nature, and carrier concentration. The poor efficiency of SnSe based cell may result from the low quality of SnSe absorber. Pure phase, good crystalline, and large grain size are required in an excellent solar cell absorber.^7^ Further studies on SnSe are expected to enhance the optoelectronic performance of SnSe material.

### 6.2 Na‐Ion Batteries and Li‐Ion Batteries

Rechargeable batteries have gained interest recently due to the increasing demand of large‐scale energy storage devices. High energy density and cycling stability distinguish the Na‐ion and Li‐ion batteries. Good performances have been reported applying SnSe in the fabrication of anode electrodes in various forms, which have been recorded in **Table** **5**.

**Table 5:** Year‐wise devolvement in the performance of SnSe‐based rechargeable batteries. PVDF: polyvinylidenefluoride binders; PAA: poly(acrylic acid); EC: ethylene carbonate; DMC: dimethyl carbonate; DEC: diethyl carbonate; MWCNT: multiwalled carbon nanotube; GO@SnSe: graphene oxide‐wrapped tin selenide nanorods composite; FEC: fluoroethylene carbonate; SnSe@CNFs: SnSe nanoparticles encapsulated in a carbon nanofibers matrix; CE: Coulombic efficiency; DC: initial discharge capacity; RC: reversible capacity

Year	Electrode		RC [mA h g^−1^]	DC [mA h g^−1^]	Stability	CE	Current Density	Ref.
	Anode	Cathode						
2006	SnSe thin films	Li	583	681	400 mA h g^−1^ (40 cycles)	–	10 µA cm^−2^	83
2011	SnSe nanocrystals, carbon black, PVDF	Li		353		43.6%	0.1 C	84
2012	SnSe nanosheets, carbon black, PVDF	Li	≈400	1009	73 mA h g^−1^ (20 cycles)	41.3%	50 mA g^−1^	85
2014	SnSe NCs Super P, PAA binder	Li	≈600	≈1200	510 mA h g^−1^ (70 cycles)	≈50%	0.1 C	86
2014	Spray‐painted SnSe NCs/carbon fabric	Li	1100	1216	676 mA h g^−1^ (80 cycles)	≈90%	200 mA g^−1^	87
2015	SnSe/C composite (nanoscale)	Na	707	≈790	Not fading (50 cycles)	75.3%	143 mA g^−1^	88
2015	SnSe/C nanocomposite, Super P, Na‐alginate binder	Na	447.7	748.5	324.9 mA h g^−1^ (200 cycles)	55.1%	500 mA g^−1^	89
2015	SnSe/C nanocomposite, Super P, Na‐alginate binder	Li	880	1097.6	633.1 mA h g^−1^ (100 cycles)	73%	500 mA g^−1^	89
2016	SnSe/MWCNT, Super P, carbon black, PVDF	Li	≈890	1241	651 mA h g^−1^ (50 cycles)	61%	40 mA g^−1^	102
2016	SnSe NPs, carbon black sodium carboxymethyl cellulose	Na	≈550	647	558 mA h g^−1^ (50 cycles)	83%	300 mA g^−1^	103
2016	GO@SnSe	Li	992	1362	764 mA h g^−1^ (100 cycles)	72%	100 mA g^−1^	104
	Bare SnSe nanorods		≈620	941	<200 (100 cycles)			
2016	SnSe@CNFs‐750, carbon black, CMC binder	Li	≈990	1148	840 mA h g^−1^ (100 cycles)	≈82%	200 mA g^−1^	90
	SnSe@CNFs‐700, carbon black, CMC binder	Li	≈860	≈1100	730 mA h g^−1^ (100 cycles)	≈82%		

Xue et al. first reported the application of SnSe thin films as anode electrode in Li‐ion batteries in 2006.^83^ The films were prepared by pulsed laser deposition. An initial discharge capacity of 681 mA h g^−1^ and a reversible discharge capacity of 583 mA h g^−1^ were reported. Performances of SnSe NCs and NSs for Na‐ion and Li‐on batteries were studied during the following eight years.^84^, ^85^, ^86^

In 2014, Wang et al. prepared a spray‐painted 3D binder‐free electrode by painting the ink based on SnSe nanocrystals onto a 3D flexible conductive carbon fabric.^87^ The electrodes exhibited a greatly enhanced reversible capacity of 676 mA h g^−1^ after 80 discharge–charge cycles at a current density of 200 mA g^−1^. The initial coulombic efficiency of 90% was the highest efficiency ever reported. The electrochemical performances of the SnSe/C composites in Na‐ion and Li‐ion batteries were further studied later by various researchers and the results were satisfactory.^88^, ^89^

In 2016, new SnSe‐based materials such as SnSe/multiwalled carbon nanotube, graphene oxide‐wrapped tin selenide nanorods composite (GO@SnSe), and SnSe nanoparticles encapsulated in a carbon nanofibers matrix (SnSe@CNFs) were reported to have better electrochemical performance in Li‐ion batteries. And batteries with GO@SnSe and SnSe@CNFs anode electrodes respectively stood out. The former exhibited the highest discharge capacities ever reported under different current densities, and a reversible capacity of 764 mA h g^−1^ after 100 cycles at the current density of 100 mA g^−1^ was delivered. The latter battery exhibited the second‐highest discharge capacities and owned better rate performance.^90^

In the aspect of Na‐ion batteries, Park et al. reported 2D SnSe nanoplates as high performance anode materials in 2016. The battery exhibited the highest discharge capacities ever reported under different current densities and a satisfactory initial coulombic efficiency (CE) of 83% was obtained.

Obviously pure SnSe material used for the anode electrode has been replaced by the composite on the basis of SnSe/C architecture. More kinds of SnSe/C or SnSe/X composites prepared for the Li‐ion and Na‐ion batteries are under study.

### 6.3 Supercapacitors

One type of flexible all‐solid‐state supercapacitors could be obtained based on SnSe materials painted onto Au‐coated polyethylene terepthalate wafers as an electrode.^87^

A polymer‐gel [polyvinylalcohol/KOH] electrolyte was utilized as both the ionic electrode and separator. The CV curves and galvanostatic charge–discharge curves evaluated in the experiment exhibited highly reversible capacity (the reversible capacity barely declines even after 2200 cycles) and high electric storage capacity, respectively. Electrochemical impedance spectroscopy was considered, indicating great performance in energy storage and high stability.

### 6.4 Phase‐Change Memory Devices

Phase‐change memory, or more precisely phase‐change random access memory, is a nonvolatile resistance variable memory technology in which the material's resistance stands for the state of the memory bit.^91^ Stacked Ge‐chalcogenide/Sn‐chalcogenide layers were prepared for these devices by Campbell and Anderson in 2006 in order to examine their capacity for resistance switching and phase‐change memory operation. Three types of stacked layers were fabricated including Ge_2_Se_3_/SnSe in the experiment. However, the result was not satisfactory. No threshold voltage was obtained in the *I*–*V* curves for the Ge_2_Se_3_/SnSe device while applying a negative potential, unless a positive potential was first taken and a proper value of the current was ensured prohibiting Joule heating effect and promising a high potential. In a word, no phase‐change behavior has been found in the negative current sweep *I*–*V* curves under standard conditions.

Considering other outstanding properties of SnSe, the trial of applying SnSe materials to the memory devices should be continued though the early experimental results are inadequate.

### 6.5 Topological Insulator

TI, a substance with special topological state, is the same as ordinary insulators in the bulk but has gapless surface or edge states. This exotic state is due to strong spin–orbit interactions and is protected by time reversal symmetry; thus if the time‐reversal symmetry is broken, TI materials will be transformed back to trivial insulators. 2D TI materials exhibit the quantum spin‐Hall effect.^92^, ^93^ Inspired by the success of TI, more and more insulating band structures have been found and studied. Very recently topological crystalline insulators (TCI) are also discovered and are reported by many authors, in TCI the topological phase is protected by the crystalline structure of material. Although TCIs share many similarities to TIs, such as insulating bulk and conducting surface, they vary in many aspects: The topology of TCIs is protected by crystal symmetries instead of time reversal symmetries in TIs. There is an even number of Dirac cones in TCIs while in TIs, this number ought to be odd. The TCIs are independent of spin–orbit interactions.^94^, ^95^

Because of the exotic properties and potential applications of these materials in electronics and spintronics, finding the materials in TCI state has opened a new era in the research. The first theoretical material to be realized as TCI is SnTe.^95^, ^96^ The related alloys Pb_1−_ *_x_*Sn*_x_*Se/Te are also confirmed to be TCIs as well.^97^, ^98^ As for SnSe materials, GeS‐type SnSe is a trivial insulator while rock‐salt SnSe is proved to be TCI theoretically, and by using Bi_2_Se_3_ as the substrate, SnSe (111) thin films have been synthesized recently. There are a number of Dirac cones at the high‐symmetry (001), (110), and (111) surfaces. The topology is protected by reflection symmetry with respect to the (110) mirror plane.^51^, ^99^

There are a few interesting characteristics of SnSe TCI materials.

I. It is well known that TCIs can be considered as the counterpart of TIs in materials without spin–orbit interactions.^94^ However, in some earlier reports, TCI materials such as Pb_1−_ *_x_*Sn*_x_*Se/Te usually possess strong spin–orbit interactions. Thus, the TCI materials with weak spin–orbit interactions, SnSe, and SnS display their importance.^99^
II. The metallic states of TIs are protected by time‐reversal symmetries while those of TCIs are supported by crystal symmetries. Nevertheless, according to Safaei et al., the surface states of SnSe and SnTe thin films grown along (111) orientation with an even number of monolayers are protected by both time‐reversal and mirror plane symmetries.^100^

## 7 Conclusion

In this work, various methods including solution and vapor deposition methods to synthesize SnSe with different morphologies including thin films, nanoflakes, nanoplates, nanosheets, etc., have been reviewed. In the study of properties, we focused on the thermoelectric and optoelectronic properties of SnSe of different morphologies—i.e., bulk, thin films, and nanostructures, and some factors affecting properties, such as metal‐doping, have also been discussed. Moreover, the SnSe‐based applications in a variety of fields like PV devices, solar cells, and solid‐state batteries have been discussed.

Conventional SnSe exhibits an anharmonic layered orthorhombic structure converting from P_nma_ to C_mcm_ when the surrounding temperature reaches around 800 K. The transition is accompanied by the decrease in the bandgap and the increase in the antibonding Sn(5s)‐anion p combination, which influences the active asymmetric density by controlling the number of the coupling of Sn(5s) and Sn(5p). A higher active asymmetric density means a higher distortion level of the electronic structure leading to the anharmonicity, which is believed to achieve low thermal conductivity at 923 K. SnSe exhibits both the direct and indirect bandgaps which are dependent on the morphology of SnSe. The appropriate bandgap value and high optical absorption coefficient make SnSe a promising material for applications in the optoelectronic fields. Moreover, SnSe‐CUB thin film has a direct bandgap of 1.4 eV which is larger than that of orthorhombic SnSe and more suitable for solar cell applications. In addition, SnS‐CUB/SnSe‐CUB stacks are also promising for futuristic applications attributing to the desirable optical bandgap value of ≈1.5 eV and higher electrical conductivity compared with SnSe‐CUB.

Although great progress has been made in the synthesis techniques, properties, and applications of SnSe material, there are some hurdles in further developing SnSe applications, some of those are discussed below.

A. High *ZT* value at 923 K is observed in SnSe single crystals. The cost for the fabrication of single crystals is enormous. It is difficult to prepare single crystalline SnSe in laboratory.
B. The defects of the synthesis of polycrystalline may lead to unexpected inhomogeneous phases. And the thermal performance of the polycrystalline SnSe is inadequate.
C. The Seebeck coefficient and electronic conductivity of SnSe materials are strongly influenced by the methods, defects, and minor impurities, which leads to the instability of the properties.
D. It is difficult to maintain the high *ZT* value in lower temperature range. Although Zhao et al. have reported *ZT* value from 0.7 to 2.0 over a broad temperature range between 300 and 773 K obtained in SnSe hole‐doped single crystals recently; however, more effective ways are still required to prohibit the decrease.

## Conflict of Interest

The authors declare no conflict of interest.
